# Barriers and enablers to engagement in participatory arts activities amongst individuals with depression and anxiety: quantitative analyses using a behaviour change framework

**DOI:** 10.1186/s12889-020-8337-1

**Published:** 2020-02-27

**Authors:** Daisy Fancourt, Louise Baxter, Fabiana Lorencatto

**Affiliations:** 1 Department of Behavioural Science and Health, University College London, 1-19 Torrington Place, London, WC1E 7HB UK; 2 Centre for Behaviour Change, University College London, London, UK

**Keywords:** Depression, Anxiety, Arts, Behaviour change, Behaviour, Barriers, Enablers

## Abstract

**Background:**

There is a large literature on the health benefits of engagement with the arts. However, there are also well-recognised challenges in ensuring equity of engagement with these activities. Specifically, it remains unclear whether individuals with poor mental health experience more barriers to participation. This study used a behaviour change framework to explore barriers to engagement in participatory arts activities amongst people with either depression or anxiety.

**Methods:**

Data were drawn from a large citizen science experiment focused on participation in creative activities. Participants who reported engaging infrequently in performing arts, visual arts, design and crafts, literature-related activities, and online, digital and electronic arts were included and categorised into no mental health problems (*n* = 1851), depression but not anxiety (*n* = 873) and anxiety but not depression (*n* = 808). Barriers and enablers to engagement were measured using an 18-item scale based on the COM-B Self-Evaluation Questionnaire, with subscales assessing psychological and physical capabilities, social and physical opportunities, and automatic and reflective motivations. Logistic regression analyses were used to identify whether individuals with either depression or anxiety reported greater barriers across any of the six domains than individuals without any mental health problems. Where differences were found, we calculated the percentage of protective association explained by various demographic, socio-economic, social, physical or geographical factors.

**Results:**

Individuals with depression and anxiety felt they would be more likely to engage in arts activities if they had greater psychological and physical capabilities, more social opportunities, and stronger automatic and reflective motivations to engage. However, they did not feel that more physical opportunities would affect their engagement. Covariates explained only 8–37% of the difference in response amongst those with and without anxiety and depression.

**Conclusions:**

Findings suggest that for individuals with poor mental health, there are certain barriers to participation that are not felt as strongly by those without any mental health problems. Mapping the behaviour change domains to potential interventions, activities that focus on increasing perceived capabilities, providing social opportunities, and reinforcing both automatic and reflective motivations to engage has the potential to help to redress the imbalance in arts participation amongst those with poor mental health.

## Background

There is a large literature on the health benefits of engagement with the arts for mental health and wellbeing [1]. However, there are also well-recognised challenges in ensuring equity of engagement with these activities. Studies have identified that some people experience more barriers to participating than others, in particular individuals of lower socio-economic status, lower educational attainment and lower income, as well as older adults and individuals from an ethnic minority group [2–7]. However, what remains less clear is whether individuals with poor mental health also experience barriers to participation above and beyond those experienced by individuals with good mental health.

As engagement with the arts is a form of human behaviour, understanding what influences engagement can be facilitated by application of theories and models of behaviour change. These theories and models represent the accumulated knowledge of what behaviour change is, what the influences on it are, and what the mechanisms of action (mediators) and moderators of change can be. There are numerous theories of behaviour change across disciplines with limited guidance for selecting one theory over another [8]. There have, therefore, been efforts within the behavioural and social sciences to synthesise theories into a minimum set of constructs representing key influences on behaviour. One such integrated theoretical model is COM-B, which posits that in order for a desired behaviour to occur, individuals must have the capability (i.e. knowledge and skills) to engage, opportunity (in their social and physical environment), and motivation (both reflective and automatic) [9].

Applying this lens, there are a number of theoretical reasons why individuals with poor mental health may experience more barriers to participation. First, in relation to *psychological capability*, individuals with poor mental health may perceive themselves to be less skilled at activities due to factors such as low self-esteem, which is bidirectionally associated with mental health [10]. Low self-esteem may lead individuals to invest less time in developing skills in arts activities and may also lead individuals to perceive the skills they do have to be inadequate. It is also possible that symptoms of specific types of mental illness may affect capabilities to engage. For example, it has been shown that depression can lead to impairments in executive functioning such as task-planning [11], while anxiety can affect concentration and lead to hyperarousal [12], both of which could reduce psychological capability to engage. Given that both depression and anxiety are correlated with poor physical health, there may be also factors affecting *physical capability*, such as illness or disability that make accessing public spaces harder, especially if community organisations do not have plans in place to facilitate access for these individuals [13, 14].

Second, in relation to *opportunities*, individuals with poor mental health are statistically more likely to experience socio-economic burden [15], which could mean that these individuals are more likely to belong to the groups identified in previous studies as facing more physical barriers to engagement due to low income, low educational attainment or living in areas with fewer arts activities to engage in. As a result, individuals with poor mental health may have fewer resources and less *physical opportunity* to engage. Further, these individuals may face barriers relating to *social opportunities.* Social exclusion has been well researched in relation to mental illness [16, 17]. Although arts activities have been found to reduce these feelings [18–20] it is possible that such feelings could act as an initial barrier to engagement. This is certainly suggested by studies focusing specifically on depression-related stigma when engaging in arts activities, which have shown that individuals with poor mental health report perceived barriers to arts engagement, such as a fear of being patronised [21].

Finally, in relation to *motivations*, individuals with poor mental health frequently have decreased participation in activities such as exercise and socialising [22–25]. One reason for this is that individuals with poor mental health may experience ‘self-stigma’; an internalising of cultural stereotypes. It has been suggested that the process of self-stigma leads to ‘behavioural futility’ - the “why try” effect [26] - and thus acts as a barrier to engaging in activities that could be good for health [27]. Further, there may also be symptoms of specific types of mental illness that affect motivations to engage. Depression can be associated with anhedonia, which can reduce automatic motivation to engage in pleasurable activities [28], while social anxiety could form a barrier to engaging in group-based activities, thereby reducing automatic and reflective motivations to engage [29].

However, these theories remain untested with data. Therefore this study used a behaviour change framework - specifically the tripartite model of capabilities, opportunities and motivations [9] - to explore what the barriers are to participatory arts engagement amongst people with poor mental health. We also tried to understand why any identified barriers might exist. First, we analysed whether the relationship between mental health and any identified barriers was explained by individual demographic, socio-economic, health or other behavioural factors. Second, we analysed whether the relationship between mental health and any identified barriers was explained by specific symptoms of mental illness by contrasting the findings for individuals with depression vs individuals with anxiety. Although depression and anxiety share non-specific components of general distress, they also have specific components, such as anhedonia in depression and physiologic arousal in anxiety [30]. As such, this comparison of findings provides a further way to understand what might be the cause of any perceived barriers amongst individuals with poor mental health.

## Methods

### Participants

**Fig. 1:**
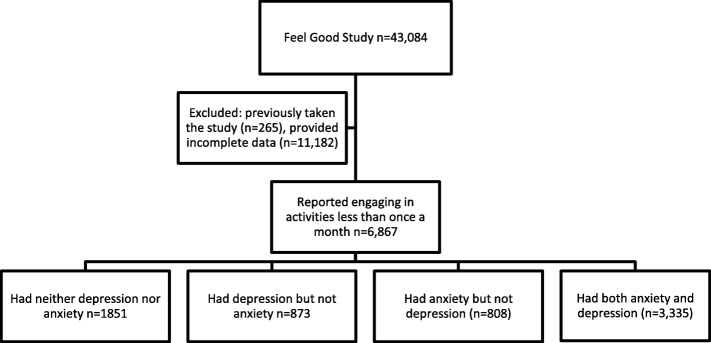
Selection of participants to the study

Data were drawn from the Feel Good data set: a sample of 43,084 individuals aged 18 and above living in the United Kingdom (UK). The data were gathered from May to June 2019 as part of a Citizen Science experiment run by the British Broadcasting Corporation (BBC) Arts. The study was promoted through the BBC Arts website as part of the UK ‘Get Creative Festival’ and individuals participated by completing an online survey that lasted approximately 20 min. For these analyses, we excluded individuals who had taken the test previously (*n* = 265), and individuals who had provided incomplete data (*n* = 11,182). As this study explored barriers to engagement, we focused on individuals who had low levels of engagement that could be indicative of experiencing barriers (whether psychological, social or physical). We therefore restricted our sample to individuals who were “infrequently” engaged (taking part in activities either on their own or with others less than once a month). This left a sample size of 6867. From this sample, we constructed four groups: individuals with neither anxiety nor depression, individuals with depression but not anxiety, individuals with anxiety but not depression, and individuals who had both anxiety and depression. We excluded this fourth group from analyses due to the challenge of attributing their barriers to either aspect of their mental health (see Fig. 1). Of the remaining 3532 participants, 873 (24.7%) individuals had depression but not anxiety, 808 (22.9%) individuals had anxiety but not depression, and 1851 (52.4%) individuals had neither anxiety nor depression.

### Measures

We focused specifically on participatory arts activities, defined in the dataset following a theorised model for population-level research as participatory activities consisting of performing arts, visual arts, design and crafts, literature-related activities, and online, digital and electronic arts [31]. Participants were asked how often they took part in any of the following activities: singing (either at home or in a choir), dancing (such as ballroom dancing/salsa classes), playing a musical instrument (either practising at home or in a band or orchestra), rehearsing or performing in a play/drama/opera/musical theatre, painting, drawing, printmaking, sculpture on your own, photography, pottery, calligraphy or jewellery making, textile crafts such as embroidery, crocheting or knitting, wood crafts such as carving or furniture making, reading a novel, stories, poetry or plays for pleasure (either alone or in a book club), creative writing, creating artworks or animations on a computer, and making films or videos. Further, in line with some previous evidence syntheses [32], we extended this definition to include gardening and baking or cooking as they are also creative activities that could be considered artistic. Although individuals’ decisions on whether or not to engage in any one specific arts activity are driven by a range of factors including perceived feelings of resonance, meaning and identity from an activity [33], engagement with the arts in general is considered to be an innate human behaviour [34]. So to allow flexibility for individual preference, we explored ‘arts activities’ as a collective.

Barriers and enablers to engagement were measured using an 18-item scale developed based on the COM-B Self-Evaluation Questionnaire [35]. Individuals were asked to select in binary form items that would encourage them to engage more frequently in arts activities, with three questions each to represent psychological capabilities, physical capabilities, social opportunities, physical opportunities, automatic motivations and reflective motivations. For example, to measure physical opportunity participants answered yes/no to the item: “In order to engage more in arts activities, I would need to have more time to do it (e.g. having time to yourself or capacity away from other commitments).” Individuals who selected any item in each of the six categories was categorised as having experienced a barrier within that category, although sensitivity analyses tested more stringent cut-offs. Overall, the scale had a Cronbach’s alpha of 0.85, with subscale alphas of 0.63 for capabilities, 0.66 for opportunities, and 0.73 for motivations. The full scale is available in the supplementary material.

Depression was measured using the 8-item Centre for Epidemiologic Studies Depression Scale (CES-D) [36]. This assesses negative affect symptoms or somatic complaints experienced in the past week using a binary reporting scale, with the total number of symptoms summed (0–8). Validation studies comparing results to diagnostic interviews have shown that a score of 3 or greater can be taken to denote the presence of mild depression, and scores of 4 or greater can be taken to denote the presence of moderate-severe depression [37, 38]. In our main analyses, individuals were categorised as “having depression” if they showed symptoms of mild, moderate or severe depression (a score of 3+), and “not having depression” if they had a score of 0–2. In our supplementary analyses, we tested moderate-severe depression more specifically, so individuals were categorised as “having depression” if they had a score of 4+ and “not having depression” if they had a score of 0–3.

Anxiety was measured using the 7-item Generalised Anxiety Disorder Questionnaire (GAD-7) [39]. This assesses how often individuals have been bothered by problems relating to anxiety in the past 2 weeks from not at all (score 0), on several days (score of 1), on more than half the days (score of 2), or nearly every day (score of 3). Scores are then summed to provide a total from 0 to 21. Scores of 5+ are considered as mild anxiety, 10+ as moderate anxiety, and 15+ as severe anxiety [39]. In our main analyses, individuals were categorised as “anxious” if they showed symptoms of mild, moderate or severe anxiety (a score of 5+), and “not anxious” if they had a score of 0–4. In our supplementary analyses, we tested moderate-severe anxiety more specifically, so individuals were categorised as “anxious” if they had a score of 10+ and “not anxious” if they had a score of 0–9.

Covariates were identified through directed acyclic graphs (DAGs) as: age (categorised as 18–30, 31–49, 50–64, and 65+), gender, ethnicity (white British vs other), employment status (in full-time employment, in part-time employment, retired or not working), educational attainment (no formal qualifications, qualifications to age 16/GCSEs/O-levels, qualifications to age 18/A-levels, degree or post-school qualifications, or postgraduate degree), household income (<£16,000, £16,000–£29,999, £30,000–£59,999, £60,000–£89,999, £90,000–£119,999, or > £120,000), type of area of dwelling (city, town, village or isolated dwelling), frequency of socialising with friends or family (once or twice a year, every few months, once or twice a month, once or twice a week, three or more times a week), physical activity (number of days in the past week in which 30 min or more of moderate or vigorous exercise has been undertaken), presence of any chronic or long-standing illness, chronic pain (none, mild, moderate or severe), presence of any problems affecting mobility, and personality (using a version of the Midlife Development Inventory which measures the five major personality traits [40]).

### Statistics

We compared demographics amongst those with and without depression and anxiety using one-way ANOVAs, Kruskal Wallis tests, and chi square tests. We then used logistic regression models to explore whether any differences held when accounting for potential explanatory factors. All model assumptions were met. In order to identify the proportion of the association explained by different factors, we built our models sequentially, calculating the percentage of protective association explained (PPAE) by the inclusion of different factors at each stage [41]. PPAE = (OR (E + C + X) – OR (E + C)) / (1 - OR (E + C)) * 100, where OR = odds ratio, E = exposure, C = covariates, and X = explanatory variable being tested.

As sensitivity analyses, we re-ran analyses using (i) covariates individually rather than grouped in categories (Supplementary Table 1), (ii) alternative cut-offs for anxiety and depression that only included those with moderate-severe depression or anxiety (Supplementary Table 2), and (iii) alternative cut-offs for COM-B components, requiring individuals to cite more than one element or to cite all three elements for it to count as an overall motivator (Supplementary Tables 3 and 4). All analyses were run in Stata v14.

## Results

### Demographics

Of the whole sample, 10.8% were aged 18–30, 43.4 were aged 31–49, 35.1% were aged 50–64, and 10.8% were aged 65+. 58.2% were female and 87.3% were of white ethnicity. All of the sample engaged with arts activities less than once a month. As a whole, the sample showed good variability across all demographic factors. There was good similarity amongst individuals with depression, anxiety, and neither in terms of ethnicity, educational attainment, the type of area in which they lived, and in certain aspects of personality type. However, there were differences in all other demographic factors (Table 1).

**Table 1:** Demographics of sample for study 1: individuals who do not engage regularly in arts activities

	Neither *n* = 1851	Individuals with depression *n* = 873	Individuals with anxiety *n* = 808	p
Age, %				**<.001**
18–30	9.6	10.0	14.6	
31–49	40.7	43.9	48.5	
50–64	36.5	36.1	30.9	
65+	13.2	10.1	5.9	
Gender, % female	55.3	56.0	67.2	**<.001**
Ethnicity, % white British	87.0	88.0	87.1	.78
Employment, %				**<.001**
In full-time employment	57	57.3	63.0	
In part-time employment	22.9	22.1	22.5	
Retired	15.5	11.5	8.2	
Not working	4.7	9.2	6.3	
Educational attainment, %				.05
No qualifications	3.7	4.0	3.8	
Qualifications to age 16	11.4	13.1	11.3	
Qualifications to age 18	12.9	14.7	14.1	
Degree/post-school qualification	45.4	44.3	40.5	
Postgraduate degree	26.6	23.9	30.3	
Household income, %				**<.001**
< £16,000	6.3	12.1	6.3	
£16,000–£29,999	17.2	20.6	15.8	
£30,000–£59,999	35.3	34.8	38.4	
£60,000–£89,999	21.9	18.9	22.9	
£90,000–£119,999	9.8	6.3	9.3	
> £120,000	9.5	7.2	7.3	
Type of area of dwelling, %				.60
City	30.6	33.8	32.2	
Town	43.7	41.7	42.1	
Village	20.8	19.8	21.8	
Isolated dwelling	4.9	4.7	4.0	
Frequency of socialising, %				**<.001**
Once or twice a year	5.8	10.1	5.0	
Every few months	17.0	21.9	18.8	
Once or twice a month	32.6	33.6	34.4	
Once or twice a week	34.3	29.9	34.5	
Three or more times a week	10.3	4.6	7.3	
Number of days exercise in past week, mean (SD)	3.9 (2.1)	3.3 (2.0)	3.7 (2.0)	**<.001**
Chronic or long-standing illness, %	11.4	18.4	13.1	**<.001**
Chronic pain, %				**<.001**
None	71.7	61.1	65.4	
Mild	21.3	27.0	24.4	
Moderate	6.0	10.1	9.4	
Severe	1.0	1.8	0.9	
Mobility problems, %	3.9	6.3	3.2	**.003**
Extravert personality, mean (SD)	10.0 (2.3)	9.7 (2.1)	10.0 (2.1)	**<.001**
Open personality, mean (SD)	11.0 (2.1)	11.0 (2.1)	11.1 (2.1)	.44
Agreeable personality, mean (SD)	11.2 (2.6)	10.9 (2.4)	10.7 (2.5)	**<.001**
Conscientious personality, mean (SD)	12.0 (1.8)	11.6 (1.9)	12.0 (1.9)	**<.001**
Neurotic personality, mean (SD)	9.5 (2.3)	9.9 (2.3)	10.9 (2.3)	**<.001**

### Capabilities

The pattern of reporting for capabilities, opportunities and motivations is shown in Table 2. 84.7% of individuals with depression, 86.3% of individuals with anxiety and 77.5% of individuals with no mental health problems reported that they would be more likely to engage in arts activities if they had greater *psychological capabilities*. These include knowing about different types of artistic activities, feeling more mentally capable of participating, and being able to make a plan for when and how to engage. Individuals with depression had a 65% higher odds of reporting psychological capabilities as a factor that would enhance engagement than individuals with no mental health problems, and individuals with anxiety had a 74% higher odds (Table 3). 21.5% of the association for depression and 36.5% of the association for anxiety was explained by factors such as personality and socio-economic status (SES) and, especially for people with depression, how frequently they engaged in physical activities. However, the association remained significant even when accounting for these factors.

**Table 2:** Percentage of individuals reporting that one or more item in each factor would encourage more engagement with arts activities

	CAPABILITIES		OPPORTUNITIES		MOTIVATIONS	
	PSYCHOLOGICAL	PHYSICAL	SOCIAL	PHYSICAL	AUTOMATIC	REFLECTIVE
Individuals with depression	**84.7%**	**90.2%**	**73.0%**	**88.0%**	**93.0%**	**88.0%**
Individuals with anxiety	**86.3%**	**91.0%**	**77.7%**	**90.2%**	**93.9%**	**90.6%**
Individuals who have neither depression nor anxiety	**77.5%**	**83.7%**	**66.1%**	**85.7%**	**89.3%**	**84.1%**

**Table 3:** Results from logistic regression analyses showing odds of reporting one or more factors that would encourage engagement in arts activities amongst individuals who do not engage regularly and have (i) depression, and (ii) anxiety, compared to individuals with neither depression or anxiety

	CAPABILITIES						OPPORTUNITIES						MOTIVATIONS					
	PSYCHOLOGICAL			PHYSICAL			SOCIAL			PHYSICAL			AUTOMATIC			REFLECTIVE		
	OR	95% CI	PPAE	OR	95% CI	PPAE	OR	95CI	PPAE	OR	95CI	PPAE	OR	95CI	PPAE	OR	95CI	PPAE
INDIVIDUALS WHO DO NOT ENGAGE: COMPARING RESPONSES OF THOSE WHO HAVE DEPRESSION (BUT NOT ANXIETY) TO THOSE WHO DO NOT HAVE DEPRESSION																		
Basic model	**1.65**	**1.32,2.05**	**–**	**1.8**	**1.39,2.33**	**–**	**1.43**	**1.19,1.71**	**–**	1.25	0.97,1.60	–	**1.63**	**1.20,2.20**	**–**	**1.41**	**1.11,1.79**	**–**
+ Demographics	**1.63**	**1.31,2.03**	**3.08%**	**1.8**	**1.39,2.33**	**0.00%**	**1.43**	**1.19,1.71**	**0.00%**	1.19	0.92,1.54		**1.58**	**1.17,2.15**	**7.94%**	**1.4**	**1.10,1.78**	**2.44%**
+ SES	**1.59**	**1.28,1.98**	**9.23%**	**1.75**	**1.35,2.27**	**6.25%**	**1.39**	**1.16,1.66**	**9.30%**	1.09	0.84,1.41		**1.6**	**1.18,2.18**	**4.76%**	**1.37**	**1.07,1.74**	**9.76%**
+ Urbanisation	**1.64**	**1.32,2.04**	**1.54%**	**1.79**	**1.38,2.31**	**1.25%**	**1.43**	**1.19,1.71**	**0.00%**	1.25	0.98,1.61		**1.62**	**1.19,2.19**	**1.59%**	**1.41**	**1.11,1.79**	**0.00%**
+ Physical health	**1.65**	**1.32,2.05**	**0.00%**	**1.66**	**1.28,2.16**	**17.50%**	**1.42**	**1.18,1.70**	**2.33%**	1.21	0.94,1.56		**1.67**	**1.23,2.27**	**−6.35%**	**1.43**	**1.12,1.82**	**−4.88%**
+ Social activity	**1.67**	**1.34,2.09**	**−3.08%**	**1.82**	**1.41,2.36**	**−2.50%**	**1.44**	**1.20,1.72**	**−2.33%**	1.2	0.93,1.54		**1.62**	**1.20,2.20**	**1.59%**	**1.43**	**1.12,1.82**	**−4.88%**
+ Physical activity	**1.59**	**1.28,1.98**	**9.23%**	**1.7**	**1.31,2.20**	**12.50%**	**1.41**	**1.18,1.69**	**4.65%**	1.17	0.91,1.51		**1.57**	**1.16,2.13**	**9.52%**	**1.4**	**1.10,1.78**	**2.44%**
+ Personality	**1.57**	**1.26,1.96**	**12.31%**	**1.72**	**1.32,2.22**	**10.00%**	**1.4**	**1.16,1.67**	**6.98%**	1.22	0.94,1.56		**1.6**	**1.18,2.17**	**4.76%**	**1.38**	**1.09,1.76**	**7.32%**
Fully-adjusted	**1.51**	**1.20,1.90**	**21.54%**	**1.51**	**1.16,1.98**	**36.25%**	**1.37**	**1.13,1.65**	**13.95%**	0.94	0.72,1.24		**1.58**	**1.15,2.18**	**7.94%**	**1.37**	**1.06,1.76**	**9.76%**
INDIVIDUALS WHO DO NOT ENGAGE: COMPARING RESPONSES OF THOSE WHO HAVE ANXIETY (BUT NOT DEPRESSION) TO THOSE WHO DO NOT HAVE ANXIETY																		
Basic model	**1.74**	**1.38,2.19**	**–**	**1.88**	**1.43,2.47**	**–**	**1.79**	**1.47,2.17**	**–**	**1.41**	**1.07,1.85**	**–**	**1.74**	**1.25,2.42**	**–**	**1.77**	**1.36,2.32**	**–**
+ Demographics	**1.62**	**1.28,2.05**	**16.22%**	**1.81**	**1.38,2.39**	**7.95%**	**1.76**	**1.45,2.14**	**3.80%**	**1.15**	**0.87,1.52**	**63.41%**	**1.55**	**1.11,2.17**	**25.68%**	**1.7**	**1.29,2.23**	**9.09%**
+ SES	**1.7**	**1.34,2.14**	**5.41%**	**1.86**	**1.42,2.45**	**2.27%**	**1.77**	**1.45,2.14**	**2.53%**	**1.22**	**0.92,1.62**	**46.34%**	**1.66**	**1.19,2.32**	**10.81%**	**1.76**	**1.34,2.31**	**1.30%**
+ Urbanisation	**1.74**	**1.38,2.19**	**0.00%**	**1.86**	**1.42,2.45**	**2.27%**	**1.79**	**1.48,2.18**	**0.00%**	**1.41**	**1.07,1.85**	**0.00%**	**1.72**	**1.24,2.39**	**2.70%**	**1.77**	**1.35,2.31**	**0.00%**
+ Physical health	**1.73**	**1.37,2.18**	**1.35%**	**1.84**	**1.40,2.42**	**4.55%**	**1.8**	**1.48,2.18**	**−1.27%**	**1.37**	**1.04,1.81**	**9.76%**	**1.76**	**1.26,2.44**	**−2.70%**	**1.8**	**1.37,2.35**	**−3.90%**
+ Social activity	**1.73**	**1.37,2.18**	**1.35%**	**1.87**	**1.42,2.45**	**1.14%**	**1.79**	**1.48,2.18**	**0.00%**	**1.38**	**1.05,1.82**	**7.32%**	**1.73**	**1.25,2.41**	**1.35%**	**1.77**	**1.35,2.31**	**0.00%**
+ Physical activity	**1.72**	**1.37,2.17**	**2.70%**	**1.84**	**1.40,2.42**	**4.55%**	**1.78**	**1.47,2.16**	**1.27%**	**1.38**	**1.05,1.81**	**7.32%**	**1.72**	**1.24,2.39**	**2.70%**	**1.77**	**1.35,2.32**	**0.00%**
+ Personality	**1.61**	**1.27,2.04**	**17.57%**	**1.81**	**1.37,2.39**	**7.95%**	**1.75**	**1.43,2.13**	**5.06%**	**1.34**	**1.01,1.78**	**17.07%**	**1.75**	**1.25,2.46**	**−1.35%**	**1.7**	**1.29,2.23**	**9.09%**
Fully-adjusted	**1.47**	**1.15,1.88**	**36.49%**	**1.63**	**1.23,2.17**	**28.41%**	**1.71**	**1.39,2.09**	**10.13%**	0.98	0.73,1.32		**1.61**	**1.14,2.29**	**17.57%**	**1.66**	**1.25,2.20**	**14.29%**

*Notes: Both analyses for depression and anxiety were run simultaneously and compared to those who had neither. Boldface = p < .05. PPAE = percentage of protective association explained. Basic model adjusted for frequency of engagement (never, once in last 12 months, twice in last 12 months, 3–4 times in last 12 months). Demographics = age, gender, ethnicity. SES = employment status, educational attainment, household income. Urbanisation = community type. Physical health = chronic illness, chronic pain, problems affecting mobility. Social activity = frequency of meeting up with friends or family. Physical activity = number of days engaged in 30 min of moderate or vigorous exercise in the past week. Personality = extraversion, openness, agreeableness, conscientiousness, neuroticism*

Further, 90.2% of individuals with depression, 91.0% of individuals with anxiety and 83.7% of individuals with no mental health problems reported that they would be more likely to engage in arts activities if they had greater *physical capabilities* (80% higher odds of reporting for individuals with depression and 88% higher odds for individuals with anxiety) (Tables 2 and 3). These include being skilled an activity, overcoming physical illness or limitations, and having sufficient energy and strength to engage. 36.3% of the association for depression and 28.4% of the association for anxiety was explained by factors such as physical health conditions, and personality. However, the association remained significant even when accounting for these factors.

### Opportunities

In relation to opportunities, 73.0% of individuals with depression, 77.7% of individuals with anxiety and 66.1% of individuals with no mental health problems reported that they would be more likely to engage in arts activities if they had greater *social opportunities* (43% higher odds of reporting for individuals with depression and 79% higher odds for individuals with anxiety) (Tables 2 and 3). These include knowing more people who engage in arts activities, having more support and encouragement from peers to engage, and feeling it is socially acceptable to engage. 14.0% of the association for depression and 10.1% of the association for anxiety was explained by factors such as SES, personality and engagement in physical activity. However, the association remained significant even when accounting for these factors.

However, individuals with depression did not report that they would be more likely to engage in arts activities if they had greater *physical opportunities*. These include having more time to engage, being able to afford the transport, resources or fees to engage, and having activities more easily accessible to engage in. 88.0% of individuals with depression, 90.2% of individuals with anxiety and 85.7% of individuals with no mental health problems reported that more physical opportunities would make them more likely to engage in arts activities (Table 2). There were higher odds of reporting physical opportunities as a factor that would enhance engagement amongst individuals with anxiety compared to individuals with no mental health problems (41% higher odds), but was attenuated by inclusion of other factors, with SES in particular explaining the initial association (Table 3).

### Motivations

In relation to motivations, 93.0% of individuals with depression, 93.9% of individuals with anxiety and 89.3% of individuals with no mental health problems reported that they would be more likely to engage in arts activities if they had greater *automatic motivations* (63% higher odds of reporting for individuals with depression and 74% higher odds for individuals with anxiety) (Table 2). These include having a habit of engaging, enjoying engaging and feeling a benefit from engaging. 7.9% of the association for depression and 17.6% of the association for anxiety was explained respectively by factors such as SES and engagement in physical activity (Table 3). However, the association remained significant even when accounting for these factors.

Finally, 88.0% of individuals with depression, 90.6% of individuals with anxiety and 84.1% of individuals with no health problems reported that they would be more likely to engage in arts activities if they had greater *reflective motivations* (41% higher odds of reporting for individuals with depression and 77% higher odds for individuals with anxiety) (Tables 2 and 3). These include believing there are benefits from engaging, having a goal to achieve, and feeling more artistic as a person. 9.8% of the association for depression and 14.3% of the association for anxiety was explained respectively by factors such as personality and SES. However, the association remained significant even when accounting for these factors.

### Sensitivity analyses

The pattern of results was maintained when restricting our definition of depression and anxiety to moderate-severe, with the exception that physical opportunities remained a significant factor reported as likely to enhance engagement amongst individuals with anxiety (but not depression) (see Supplementary Table 2). Results were also consistent when applying more stringent cut-offs to the number of items within each factor that had to be selected for that factor to count as a behavioural motivator to engagement (see Supplementary Tables 3 and 4). The only exception was that the finding for physical capabilities for individuals with depression was entirely explained when requiring participants to report all three items within that factor for it to be considered a barrier to participation.

## Discussion

This study found that individuals with depression and anxiety felt they would be more likely to engage in arts activities if they had greater psychological and physical capabilities, more social opportunities, and stronger automatic and reflective motivations to engage. However, they did not feel that more physical opportunities would affect their engagement. In considering which factors could be addressed to support greater engagement amongst individuals with poor mental health, we mapped our findings to the Behaviour Change Wheel (a framework of behavioural intentions) [9, 35], and a taxonomy of 93 Behaviour Change Techniques [42]. These mappings pair behavioural influences (i.e. the COM-B model dimensions of Capability, Opportunity, Motivation), with the types of behaviour change interventions and techniques that are likely to be relevant and effective to targeting identified influences on the behaviour of interest. This in turn provides a systematic basis for moving from ‘behavioural diagnosis’ of barriers/enablers to selecting intervention strategies to overcome these, which are discussed more below.

Overall, these findings suggest that for individuals with poor mental health, there are certain barriers to participation that are not felt as strongly by those without mental health problems. People with depression and anxiety both reported that enhanced feelings of *capability* would encourage them to engage more with arts activities. This suggests that, compared to people without mental health problems, these individuals feel less *psychologically capable* of engaging (e.g. they know less about different types of activities available, feel less mentally capable of engaging, or are less confident in making plans for when and how to engage) and less *physically capable* (e.g. they feel less skilled in specific arts activities, feel they have physical limitations to overcome, or feel they lack energy or strength to engage). Personality explained the largest amount of this difference, in particular levels of conscientiousness in people with depression and levels of neuroticism in both groups. Our analyses showed that individuals with depression had lower levels of conscientiousness and both groups had higher levels of neuroticism than people without mental health problems. So adjusting for personality helped to explain some of the difference in capacity. This builds on previous research which has shown how personality traits such as conscientiousness have been associated with better mental health and with aspects of psychological capability such as self-efficacy [43, 44]. Our results also suggest that people with depression and anxiety have lower perceived *physical capability* to engage. This was partly explained by differences in physical health, in particular higher levels chronic illness and chronic pain in people with poor mental health, and lower levels of physical activity in people with depression. However, it is notable that the differences in whether perceived capability would affect engagement in arts activities remained independent of factors such as demographics, personality and physical health. This suggests that, regardless of differences in these factors between those with and without mental health problems, depression and anxiety themselves could lead to reduced perceptions of capability, with these perceptions acting as manifestations of their mental health conditions. In considering interventions that could help address these barriers, a combination of training and enabling activities that initially engage individuals through taster sessions or demonstrations and then encourage individuals through graded tasks and positive feedback could be explored in future studies to assess if these approaches can help to enhance feelings of capability (Table 4).

**Table 4:** Behaviour change techniques and example strategies for removing barriers to engaging in specific arts activities amongst individuals with depression

COM-B component	Example of relevant barriers to engagement in arts activities	Intervention type^a^	Behaviour Change Techniques^b^	Example strategy to encourage engagement in arts activities
Psychological Capability	Knowing less about different types of activities available, feeling less mentally capable to engage, and being less confident in making plans for when and how to engage	Education Enablement	Instruction on how to perform the behaviour Action planning Graded tasks	Educational resources (i.e. leaflet, websites, helplines) outlining the range of activities available in the local area and their dates and times. An action planning template/tool to prompt individuals to formulate a plan for which activities they will attend, where, when, on which days, how often. Encouragement to individuals to start small- formulating a plan to engage in one or two activities at a low frequency, and gradually increase number of activities/ frequency as appropriate
Physical Capability	Feeling less skilled in specific arts activities, having physical limitations affecting participation, or having less energy or strength	Training	Instruction on how to perform behaviour Demonstration of the behaviour AND/OR Behavioural practice rehearsal Feedback on behaviour	Educational resources (i.e. leaflet, websites, helplines) outlining the range of activities available in the local area and their dates and times. Taster sessions, or drop-in training sessions, for different activities, in which individuals can receive instructions and tuition on the ‘basics’ or a specific skill they are struggling with, watch demonstrations of it being performed Practice engaging in activities under supervision (e.g. in a class) with opportunity for feedback and correction as needed
Social Opportunity	Not knowing people who engage in arts activities, feeling unsupported by friends or family, or feeling participation is not socially acceptable	Modelling Persuasion Enablement	Credible Source Demonstration of the behaviour Information about others’ approval Social practical support	Campaigns or resources (leaflets, websites), or social media, featuring testimonials of people with depression and anxiety modelling engagement in arts activities and advocating benefits associated with this AND/OR healthcare professionals endorsing it AND/OR testimonial from friends and families also endorsing the benefits of a loved one engaging in arts activities. Taster sessions, or drop-in training sessions, for different activities, in which individuals can receive instructions and tuition on the ‘basics’ or a specific skill they are struggling with, watch demonstrations of it being performed Opportunities for performances or showcase events for friends and family to see the product of the activities and give direct feedback Tips or direct help from individuals to seek social support from others (i.e. peers, friends and family members) to engage in artistic activities. This could be identifying joint activities they can do with others, providing reminders, verbal encouragement, support getting to/from activities if needed.
Physical Opportunity	Not having sufficient time to engage, resources to engage, or easily accessible activities	Incentives	Material incentive Practical incentive AND/OR enablement	Provision of free activities, vouchers or financial discounts for participating in artistic activities Direct referral or recommendation to attend a specific activity from a health or social care professional, with follow up on attendance and experience in the activity
Automatic Motivation	Not having a habit of engaging, not enjoying engaging, and not feeling a benefit from engaging	Persuasion Education	Self-monitoring Information about consequences (health, social, environmental) Credible source	Provision of opportunity for individuals to reflect on their experience after engaging in an artistic activity e.g. providing a diary or rating scale asking individuals to state something they enjoyed from engaging in the activity. Educational materials and resources outlining the different types of benefits of engaging in creative activities, and the evidence base for these e.g. research evidence and/or testimonials from credible/relatable individuals. Campaigns or resources (leaflets, websites), or social media, featuring testimonials of people with depression and anxiety modelling engagement in arts activities and advocating benefits associated with this AND/OR healthcare professionals endorsing it AND/OR testimonial from friends and families also endorsing the benefits of a loved one engaging in arts activities.
Reflective Motivation	Not believing there are personal benefits from engaging, not having a goal to achieve, and not feeling artistic or imaginative as a person	Persuasion Modelling	Self-monitoring Identity associated with behaviour change Goal setting Credible source Demonstration	Encouragement for individuals to track changes in their health and wellbeing over a period of engagement e.g. through diaries, rating scales before and after engagement Encouragement for individuals to track changes in their identity, e.g. through keeping a diary, reflecting on and affirming/verbalising positive changes relating to being creative and artistic. Setting of goals to achieve e.g. performances or exhibitions to take part in, work to produce or skills to learn Campaigns or resources (leaflets, websites), or social media, featuring testimonials of people who may not identify as especially ‘artistic’ advocating benefits associated with engagement Taster sessions, or drop-in training sessions, for different activities, to normalise the creativity or artistic credentials needed to engage

^a^Intervention type labels from the Behaviour Change Wheel (Michie et al. 2011); ^b^Behaviour Change Technique (BCT) labels from BCT Taxonomy v1 (Michie et al. 2013)

In relation to opportunities, there were more mixed results. There was little evidence that individuals with anxiety or depression experience fewer physical opportunities to engage. But compared to people without mental health problems, people with poor mental health appear to have fewer social opportunities to engage (e.g. they know fewer people who engage in arts activities, they feel less support and encouragement from peers to engage, or they feel it is less socially acceptable to engage). For depression, this difference in social opportunities was partly explained by socio-economic factors, suggesting that lower levels of wealth, education and employment could reduce an individual’s social network who might support such engagement. However, noticeably these individuals were not just more isolated, as social activities did not explain away the association. Therefore, it appears that having a social network *that engages in such activities* is important, beyond just having a social network itself. Whether individuals lived in a more urban or more rural area did not explain any of the association. This suggests that there was not simply a difference in area of dwelling guiding engagement more in people with poor mental health than without (e.g. through more activities being available in urban than rural locations). For both anxiety and depression, the differences in whether perceived social opportunity would affect engagement in arts activities persisted independent of identified explanatory factors. In considering why this might be, it is relevant to explore whether people with poor mental health feel it is less socially acceptable for them to engage. Our results suggest that there was no direct discrimination based on demographic factors such as age, gender or ethnicity as consideration of these factors did not attenuate the difference in results for social opportunities, but previous research has suggested that stigma is a key barrier to arts engagement [21]. In considering interventions that could help address these barriers, modelling interventions such as the endorsement of activities by healthcare professionals alongside enabling interventions that provide feedback to individuals that people around them approve of their engagement could be explored in future studies to assess if these approaches can improve social opportunities to engage (Table 4).

In relation to motivations, people with poor mental health appear to have lower automatic motivations to engage in arts activities (such as less strong habits of engaging, lower enjoyment from engaging, or fewer perceived benefits from engaging) and lower reflective motivations to engage (e.g. lower beliefs in the benefits of engaging, less strong goals from engaging or less strong identities as an artistic person). Notably, whether individuals engaged in other social activities had very small associations with their motivations to engage in artistic activities. This suggests that engagement in social activities and arts activities are quite distinct and resonates with research showing independent associations between both social engagement and arts and cultural engagement and various mental and physical health outcomes [45–47]. Demographic and socio-economic factors explained some of the association, with young people and those who were unemployed less likely to report strong motivations to engage. However, the differential associations between those with and without mental health problems persisted independently. In considering interventions that could help address these barriers, a combination of persuasion activities such as self-monitoring of engagement so that individuals record if they have enjoyed activities, and education activities such as providing resources on the benefits of engagement for mental health could be explored in future studies to assess if these approaches can help to enhance feelings of capability (Table 4).

This study has a number of strengths. It used a theoretical framework to consider what the differences in capabilities, opportunities and motivations to engagement are in individuals with poor mental health. Our measures of depression and anxiety were validated and findings were consistent when applying different cut-off thresholds. Further, we were able to identify to what extent specific demographic, socio-economic, health-related or personality-based factors explained our findings, showing persistent differences regardless of all of these factors. However, there were several limitations. First, this sample was not nationally representative, although our sample was large and had good distribution across different factors. Second, we focused on behavioural *intentions*. This suggests that if certain factors could be addressed, people with poor mental health could be encouraged to engage more in arts activities. However, whether this would lead to altered patterns of behaviour would need to be explored in future studies. Relatedly, it is possible that some of the perceived barriers reported by individuals with anxiety and depression could in fact be manifestations of their mental health conditions. For psychological capabilities and both reflective and automatic motivations, this is likely the case and is already discussed, with the proposed interventions aimed at supporting these individuals to feel more capable and motivated to engage whilst taking into account any symptoms of mental health conditions being experienced. But for physical capabilities or social opportunities, reported barriers may in fact be due to more negative reporting from individuals with anxiety or depression. However, it should be noted that there were no differences in physical opportunity barriers between those with poor mental health compared to those without once adjusting for demographic and socio-economic factors. This suggests that individuals were not merely reporting more negatively across all domains.

## Conclusion

Therefore, in concluding, this study showed that there are specific patterns of capabilities, opportunities and motivations that could influence participation in arts activities amongst individuals with depression and anxiety and proposes interventions that focus on increasing perceived psychological and physical capabilities, providing social opportunities, and reinforcing both automatic and reflective motivations to engage. Given the breadth of research showing the benefits of arts activities for improving symptoms of depression and anxiety and enhancing wellbeing, future studies are encouraged to explore whether behaviour change interventions could reduce inequities in participation.

## Supplementary information

**Additional file 1:** Questions on arts behavioural intentions. **Table S1.** Results from logistic regression analyses showing odds of reporting one or more factors that would encourage engagement in artistic hobbies amongst individuals who do not engage regularly and have (i) depression, and (ii) anxiety, compared to individuals with neither depression or anxiety [showing all predictors individually]. **Figure S1.** Selection of participants to the study (moderate-severe depression and anxiety). **Table S2.** Results from logistic regression analyses showing odds of reporting one or more factors that would encourage engagement in artistic hobbies amongst individuals with (i) moderate-severe depression, and (ii) moderate-severe anxiety compared to individuals with neither moderate-severe depression nor moderate-severe anxiety. **Table S3.** Results from logistic regression analyses showing odds of reporting two or more factors that would encourage engagement in artistic hobbies amongst individuals with (i) depression, and (ii) anxiety compared to individuals with neither depression or anxiety. **Table S4.** Results from logistic regression analyses showing odds of reporting three or more factors that would encourage engagement in artistic hobbies amongst individuals with (i) depression, and (ii) anxiety compared to individuals with neither depression or anxiety.

## Data Availability

The datasets analysed during the current study are available from the corresponding author on reasonable request.

## Abbreviations

ANOVA: Analysis of Variance
BBC: British Broadcasting Corporation
CEDS: Centre for Epidemiological Studies Depression Scale
DAGs: Directed Acyclic Graphs
GAD – 7: 7 item Generalised Anxiety Disorder Questionnaire
OR: Odds Ratio
PPAE: Percentage of Protective Association Explained
SES: Socio-economic Status
UK: United Kingdom
