# Supplementary material for: Barriers and enablers to engagement in participatory arts activities amongst individuals with depression and anxiety: quantitative analyses using a behaviour change framework
Source: BMC Public Health. 2020 Feb 27;20:272. doi: 10.1186/s12889-020-8337-1 (PMC7045421; doi:10.1186/s12889-020-8337-1)
Supplement: Supplementary file 1 — Additional file 1: Questions on arts behavioural intentions. Table S1. Results from logistic regression analyses showing odds of reporting one or more factors that would encourage engagement in artistic hobbies amongst individuals who do not engage regularly and have (i) depression, and (ii) anxiety, compared to individuals with neither depression or anxiety [showing all predictors individually]. Figure S1. Selection of participants to the study (moderate-severe depression and anxiety). Table S2. Results from logistic regression analyses showing odds of reporting one or more factors that would encourage engagement in artistic hobbies amongst individuals with (i) moderate-severe depression, and (ii) moderate-severe anxiety compared to individuals with neither moderate-severe depression nor moderate-severe anxiety. Table S3. Results from logistic regression analyses showing odds of reporting two or more factors that would encourage engagement in artistic hobbies amongst individuals with (i) depression, and (ii) anxiety compared to individuals with neither depression or anxiety. Table S4. Results from logistic regression analyses showing odds of reporting three or more factors that would encourage engagement in artistic hobbies amongst individuals with (i) depression, and (ii) anxiety compared to individuals with neither depression or anxiety. [file 12889_2020_8337_MOESM1_ESM.docx]

Supplementary Material

**Questions on arts behavioural intentions**

*All rated ‘strongly disagree’ to ‘strongly agree’*

What do you think it would take for you to engage more in artistic activities? (Some of the items may look strange, but that is just because we need to include anything that might possibly apply for some people.)

In order to engage more in artistic hobbies I would need to:

Psychological capability

1. Know more about different types of artistic activities (e.g. the breadth of activities you could take up)
2. Feel more mentally capable (e.g. have more belief in myself, feel more positive, have better concentration)
3. Make a plan for when and how to engage e.g. (find a class, clear time in my diary)

Physical capability

1. Be more skilled at the activity (e.g. be able to play the piano or use knitting needles)
2. Overcome physical limitations (e.g. find a way around physical barriers to taking part caused by an illness or disability)
3. Have more energy or strength (e.g. not be too tired after work)

Social opportunity

1. Know more people who do the activity (e.g. see friends, neighbours or colleagues being artistic)
2. Have more support from others (e.g. friends or a group who encourage me to engage)
3. Feel it’s socially acceptable for me to engage (e.g. not feel unwelcome due to my age/ gender/ cultural background)

Physical opportunity

1. Have more time to do it (e.g. having time to yourself or capacity away from other commitments)
2. Be able to afford the resources (e.g. have the money to pay for transport, lessons, books or art supplies)
3. Have activities more easily accessible (e.g. have a class closer to my home or space in my own home)

Automatic motivation

1. Have a habit of doing it (e.g. have a weekly class)
2. Enjoy engaging in artistic activities (e.g. feel a sense of pleasure or satisfaction from it)
3. Feel more benefit when I engage (e.g. lower stress or better mood)

Reflective motivation

1. Believe that it would be good for me (e.g. know that a artistic activity could help me overcome a physical or mental health problem or make me more employable)
2. Have a goal to achieve (e.g. a performance or exhibition or a finished item to prepare for)
3. Feel more artistic as a person (e.g. feel artistic or imaginative)

**Supplementary Table 1: Results from logistic regression analyses showing odds of reporting one or more factors that would encourage engagement in artistic hobbies amongst individuals who do not engage regularly and have (i) depression, and (ii) anxiety, compared to individuals with neither depression or anxiety [showing all predictors individually]**

|  | CAPABILITIES | | | | | | OPPORTUNITIES | | | | | | MOTIVATORS | | | | | |  |
| --- | --- | --- | --- | --- | --- | --- | --- | --- | --- | --- | --- | --- | --- | --- | --- | --- | --- | --- | --- |
|  | **PSYCHOLOGICAL** | | | **PHYSICAL** | | | **SOCIAL** | | | **PHYSICAL** | | | **AUTOMATIC** | | | **REFLECTIVE** | | |  |
|  | **OR** | **95% CI** | **PPAE** | **OR** | **95% CI** | **PPAE** | **OR** | **95CI** | **PPAE** | **OR** | **95CI** | **PPAE** | **OR** | **95CI** | **PPAE** | **OR** | **95CI** | **PPAE** |  |
| INDIVIDUALS WHO DO NOT ENGAGE: COMPARING RESPONSES OF THOSE WHO ARE DEPRESSED (BUT NOT ANXIOUS) TO THOSE WHO ARE NOT DEPRESSED | | | | | | | | | | | | | | | | | | | |
| Unadjusted | **1.65** | **1.32,2.05** | **-** | **1.8** | **1.39,2.33** | **-** | **1.43** | **1.19,1.71** | **-** | 1.25 | 0.97,1.60 | - | **1.63** | **1.20,2.20** | **-** | **1.41** | **1.11,1.79** | **-** |  |
| + Age | **1.63** | **1.31,2.02** | **3.08%** | **1.8** | **1.39,2.33** | **0.00%** | **1.42** | **1.19,1.70** | **2.33%** | 1.19 | 0.93,1.54 | - | **1.58** | **1.16,2.14** | **7.94%** | **1.39** | **1.09,1.77** | **4.88%** |  |
| + Gender | **1.64** | **1.32,2.05** | **1.54%** | **1.8** | **1.39,2.33** | **0.00%** | **1.43** | **1.19,1.71** | **0.00%** | 1.25 | 0.97,1.60 | - | **1.62** | **1.20,2.20** | **1.59%** | **1.41** | **1.11,1.79** | **0.00%** |  |
| + Ethnicity | **1.65** | **1.33,2.06** | **0.00%** | **1.81** | **1.40,2.33** | **-1.25%** | **1.43** | **1.20,1.71** | **0.00%** | 1.25 | 0.98,1.61 | - | **1.63** | **1.21,2.21** | **0.00%** | **1.41** | **1.11,1.80** | **0.00%** |  |
| + Employment | **1.63** | **1.31,2.02** | **3.08%** | **1.78** | **1.38,2.30** | **2.50%** | **1.41** | **1.18,1.69** | **4.65%** | 1.19 | 0.92,1.53 | - | **1.59** | **1.17,2.15** | **6.35%** | **1.4** | **1.10,1.78** | **2.44%** |  |
| + Education | **1.65** | **1.32,2.05** | **0.00%** | **1.8** | **1.39,2.33** | **0.00%** | **1.42** | **1.19,1.70** | **2.33%** | 1.24 | 0.97,1.60 | - | **1.65** | **1.22,2.24** | **-3.17%** | **1.41** | **1.11,1.79** | **0.00%** |  |
| + Wealth | **1.62** | **1.30,2.02** | **4.62%** | **1.78** | **1.37,2.30** | **2.50%** | **1.4** | **1.17,1.68** | **6.98%** | 1.21 | 0.94,1.56 | - | **1.67** | **1.23,2.27** | **-6.35%** | **1.39** | **1.09,1.77** | **4.88%** |  |
| + Geography | **1.64** | **1.32,2.04** | **1.54%** | **1.79** | **1.38,2.31** | **1.25%** | **1.43** | **1.19,1.71** | **0.00%** | 1.25 | 0.98,1.61 | - | **1.62** | **1.19,2.19** | **1.59%** | **1.41** | **1.11,1.79** | **0.00%** |  |
| + Illness | **1.66** | **1.33,2.06** | **-1.54%** | **1.71** | **1.32,2.21** | **11.25%** | **1.42** | **1.19,1.70** | **2.33%** | 1.25 | 0.97,1.61 | - | **1.66** | **1.22,2.25** | **-4.76%** | **1.42** | **1.11,1.80** | **-2.44%** |  |
| + Pain | **1.64** | **1.31,2.04** | **1.54%** | **1.7** | **1.32,2.21** | **12.50%** | **1.42** | **1.19,1.71** | **2.33%** | 1.2 | 0.93,1.54 | - | **1.66** | **1.22,2.25** | **-4.76%** | **1.42** | **1.12,1.81** | **-2.44%** |  |
| + Mobility | **1.66** | **1.33,2.06** | **-1.54%** | **1.76** | **1.36,2.27** | **5.00%** | **1.42** | **1.19,1.70** | **2.33%** | 1.24 | 0.97,1.59 | - | **1.64** | **1.21,2.23** | **-1.59%** | **1.41** | **1.11,1.80** | **0.00%** |  |
| + Socialising | **1.67** | **1.34,2.09** | **-3.08%** | **1.82** | **1.41,2.36** | **-2.50%** | **1.44** | **1.20,1.72** | **-2.33%** | 1.2 | 0.93,1.54 | - | **1.62** | **1.20,2.20** | **1.59%** | **1.43** | **1.12,1.82** | **-4.88%** |  |
| + Exercise | **1.59** | **1.28,1.98** | **9.23%** | **1.7** | **1.31,2.20** | **12.50%** | **1.41** | **1.18,1.69** | **4.65%** | 1.17 | 0.91,1.51 | - | **1.57** | **1.16,2.13** | **9.52%** | **1.4** | **1.10,1.78** | **2.44%** |  |
| + Extraversion | **1.67** | **1.34,2.08** | **-3.08%** | **1.78** | **1.37,2.30** | **2.50%** | **1.43** | **1.20,1.71** | **0.00%** | 1.24 | 0.96,1.59 | - | **1.62** | **1.20,2.19** | **1.59%** | **1.43** | **1.12,1.81** | **-4.88%** |  |
| + Openness | **1.65** | **1.32,2.05** | **0.00%** | **1.8** | **1.40,2.33** | **0.00%** | **1.43** | **1.19,1.71** | **0.00%** | 1.25 | 0.97,1.61 | - | **1.63** | **1.20,2.20** | **0.00%** | **1.41** | **1.11,1.80** | **0.00%** |  |
| + Agreeableness | **1.65** | **1.32,2.05** | **0.00%** | **1.81** | **1.40,2.34** | **-1.25%** | **1.43** | **1.20,1.72** | **0.00%** | 1.25 | 0.98,1.61 | - | **1.62** | **1.20,2.20** | **1.59%** | **1.41** | **1.11,1.79** | **0.00%** |  |
| + Conscientiousness | **1.59** | **1.28,1.98** | **9.23%** | **1.76** | **1.36,2.27** | **5.00%** | **1.4** | **1.17,1.68** | **6.98%** | 1.25 | 0.97,1.60 | - | **1.6** | **1.18,2.17** | **4.76%** | **1.38** | **1.09,1.76** | **7.32%** |  |
| + Neuroticism | **1.61** | **1.30,2.01** | **6.15%** | **1.78** | **1.38,2.30** | **2.50%** | **1.42** | **1.18,1.69** | **2.33%** | 1.23 | 0.96,1.58 | - | **1.63** | **1.20,2.21** | **0.00%** | **1.39** | **1.09,1.77** | **4.88%** |  |
|  |  |  |  |  |  |  |  |  |  |  |  |  |  |  |  |  |  |  |  |
| INDIVIDUALS WHO DO NOT ENGAGE: COMPARING RESPONSES OF THOSE WHO ARE ANXIOUS (BUT NOT DEPRESSED) TO THOSE WHO ARE NOT ANXIOUS | | | | | | | | | | | | | | | | | | | |
| Basic model | **1.74** | **1.38,2.19** | **-** | **1.88** | **1.43,2.47** | **-** | **1.79** | **1.47,2.17** | **-** | **1.41** | **1.07,1.85** | - | **1.74** | **1.25,2.42** | **-** | **1.77** | **1.36,2.32** | **-** |  |
| + Age | **1.66** | **1.32,2.10** | **10.81%** | **1.84** | **1.40,2.42** | **4.55%** | **1.74** | **1.43,2.12** | **6.33%** | 1.23 | 0.93,1.62 |  | **1.59** | **1.14,2.21** | **20.27%** | **1.71** | **1.31,2.25** | **7.79%** |  |
| + Gender | **1.68** | **1.33,2.12** | **8.11%** | **1.85** | **1.41,2.43** | **3.41%** | **1.79** | **1.48,2.18** | **0.00%** | **1.32** | **1.00,1.74** | **21.95%** | **1.69** | **1.21,2.35** | **6.76%** | **1.75** | **1.33,2.29** | **2.60%** |  |
| + Ethnicity | **1.75** | **1.38,2.20** | **-1.35%** | **1.88** | **1.43,2.47** | **0.00%** | **1.79** | **1.48,2.18** | **0.00%** | **1.41** | **1.07,1.86** | **0.00%** | **1.75** | **1.26,2.42** | **-1.35%** | **1.78** | **1.36,2.33** | **-1.30%** |  |
| + Employment | **1.71** | **1.36,2.16** | **4.05%** | **1.86** | **1.42,2.45** | **2.27%** | **1.79** | **1.47,2.17** | **0.00%** | 1.28 | 0.97,1.68 |  | **1.66** | **1.19,2.31** | **10.81%** | **1.75** | **1.34,2.29** | **2.60%** |  |
| + Education | **1.74** | **1.38,2.20** | **0.00%** | **1.91** | **1.45,2.50** | **-3.41%** | **1.79** | **1.47,2.17** | **0.00%** | **1.39** | **1.06,1.83** | **4.88%** | **1.76** | **1.26,2.44** | **-2.70%** | **1.81** | **1.38,2.36** | **-5.19%** |  |
| + Wealth | **1.73** | **1.37,2.19** | **1.35%** | **1.87** | **1.42,2.45** | **1.14%** | **1.78** | **1.47,2.16** | **1.27%** | **1.4** | **1.06,1.84** | **2.44%** | **1.74** | **1.25,2.42** | **0.00%** | **1.76** | **1.35,2.30** | **1.30%** |  |
| + Geography | **1.74** | **1.38,2.19** | **0.00%** | **1.86** | **1.42,2.45** | **2.27%** | **1.79** | **1.48,2.18** | **0.00%** | **1.41** | **1.07,1.85** | **0.00%** | **1.72** | **1.24,2.39** | **2.70%** | **1.77** | **1.35,2.31** | **0.00%** |  |
| + Illness | **1.74** | **1.38,2.20** | **0.00%** | **1.86** | **1.41,2.44** | **2.27%** | **1.79** | **1.47,2.17** | **0.00%** | **1.41** | **1.07,1.85** | **0.00%** | **1.75** | **1.26,2.43** | **-1.35%** | **1.78** | **1.36,2.32** | **-1.30%** |  |
| + Pain | **1.74** | **1.38,2.19** | **0.00%** | **1.82** | **1.39,2.39** | **6.82%** | **1.79** | **1.47,2.17** | **0.00%** | **1.37** | **1.04,1.81** | **9.76%** | **1.76** | **1.27,2.45** | **-2.70%** | **1.8** | **1.37,2.35** | **-3.90%** |  |
| + Mobility | **1.74** | **1.38,2.19** | **0.00%** | **1.89** | **1.44,2.48** | **-1.14%** | **1.79** | **1.48,2.17** | **0.00%** | **1.41** | **1.07,1.85** | **0.00%** | **1.74** | **1.25,2.41** | **0.00%** | **1.77** | **1.36,2.32** | **0.00%** |  |
| + Socialising | **1.73** | **1.37,2.18** | **1.35%** | **1.87** | **1.42,2.45** | **1.14%** | **1.79** | **1.48,2.18** | **0.00%** | **1.38** | **1.05,1.82** | **7.32%** | **1.73** | **1.25,2.41** | **1.35%** | **1.77** | **1.35,2.31** | **0.00%** |  |
| + Exercise | **1.72** | **1.37,2.17** | **2.70%** | **1.84** | **1.40,2.42** | **4.55%** | **1.78** | **1.47,2.16** | **1.27%** | **1.38** | **1.05,1.81** | **7.32%** | **1.72** | **1.24,2.39** | **2.70%** | **1.77** | **1.35,2.32** | **0.00%** |  |
| + Extraversion | **1.74** | **1.38,2.19** | **0.00%** | **1.88** | **1.43,2.47** | **0.00%** | **1.79** | **1.47,2.17** | **0.00%** | **1.41** | **1.07,1.85** | **0.00%** | **1.74** | **1.25,2.42** | **0.00%** | **1.78** | **1.36,2.32** | **-1.30%** |  |
| + Openness | **1.74** | **1.38,2.19** | **0.00%** | **1.88** | **1.43,2.47** | **0.00%** | **1.79** | **1.47,2.17** | **0.00%** | **1.4** | **1.06,1.84** | **2.44%** | **1.73** | **1.25,2.41** | **1.35%** | **1.78** | **1.36,2.33** | **-1.30%** |  |
| + Agreeableness | **1.74** | **1.38,2.20** | **0.00%** | **1.89** | **1.44,2.48** | **-1.14%** | **1.8** | **1.49,2.19** | **-1.27%** | **1.41** | **1.08,1.86** | **0.00%** | **1.74** | **1.25,2.41** | **0.00%** | **1.77** | **1.36,2.32** | **0.00%** |  |
| + Conscientiousness | **1.75** | **1.39,2.20** | **-1.35%** | **1.88** | **1.43,2.47** | **0.00%** | **1.79** | **1.48,2.17** | **0.00%** | **1.41** | **1.07,1.85** | **0.00%** | **1.74** | **1.25,2.42** | **0.00%** | **1.78** | **1.36,2.32** | **-1.30%** |  |
| + Neuroticism | **1.61** | **1.27,2.04** | **17.57%** | **1.8** | **1.36,2.38** | **9.09%** | **1.74** | **1.42,2.11** | **6.33%** | **1.34** | **1.01,1.77** | **17.07%** | **1.76** | **1.26,2.46** | **-2.70%** | **1.7** | **1.29,2.23** | **9.09%** |  |

*Notes: Both depressed and anxious analyses run simultaneously and compared to those who are not depressed or anxious. Boldface=p<.05. PPAE=percentage of protective association explained. Basic model adjusted for frequency of engagement (never, once in last 12 months, twice in last 12 months, 3-4 times in last 12 months).*

**Supplementary Figure 1: Selection of participants to the study (moderate-severe depression and anxiety)**

**Supplementary Table 2: Results from logistic regression analyses showing odds of reporting one or more factors that would encourage engagement in artistic hobbies amongst individuals with (i) moderate-severe depression, and (ii) moderate-severe anxiety compared to individuals with neither moderate-severe depression nor moderate-severe anxiety**

|  | CAPABILITIES | | | | | | OPPORTUNITIES | | | | | | MOTIVATORS | | | | | |
| --- | --- | --- | --- | --- | --- | --- | --- | --- | --- | --- | --- | --- | --- | --- | --- | --- | --- | --- |
|  | **PSYCHOLOGICAL** | | | **PHYSICAL** | | | **SOCIAL** | | | **PHYSICAL** | | | **AUTOMATIC** | | | **REFLECTIVE** | | |
|  | **OR** | **95% CI** | **PPAE** | **OR** | **95% CI** | **PPAE** | **OR** | **95CI** | **PPAE** | **OR** | **95CI** | **PPAE** | **OR** | **95CI** | **PPAE** | **OR** | **95CI** | **PPAE** |
| DEPRESSED, NOT ANXIOUS | | | | | | | | | | | | | | | | | | |
| Basic model | **2.37** | **1.98,2.84** | - | **2.24** | **1.82,2.77** | **-** | **1.73** | **1.51,1.99** | **-** | **1.43** | **1.18,1.73** | **-** | **2.07** | **1.61,2.66** | **-** | **1.95** | **1.60,2.39** | **-** |
| + Demographics | **2.33** | **1.94,2.80** | **2.92%** | **2.22** | **1.80,2.74** | **1.61%** | **1.72** | **1.50,1.98** | **1.37%** | **1.37** | **1.12,1.66** | **13.95%** | **2** | **1.56,2.58** | **6.54%** | **1.93** | **1.58,2.36** | **6.54%** |
| + SES | **2.25** | **1.87,2.70** | **8.76%** | **2.17** | **1.76,2.69** | **5.65%** | **1.66** | **1.45,1.91** | **9.59%** | **1.26** | **1.04,1.54** | **39.53%** | **2.04** | **1.58,2.63** | **2.80%** | **1.9** | **1.55,2.33** | **2.80%** |
| + Urbanisation | **2.37** | **1.97,2.84** | **0.00%** | **2.23** | **1.80,2.75** | **0.81%** | **1.73** | **1.50,1.98** | **0.00%** | **1.42** | **1.18,1.72** | **2.33%** | **2.05** | **1.59,2.63** | **1.87%** | **1.94** | **1.59,2.37** | **1.87%** |
| + Physical health | **2.36** | **1.96,2.84** | **0.73%** | **2.07** | **1.67,2.56** | **13.71%** | **1.73** | **1.51,1.99** | **0.00%** | **1.41** | **1.16,1.71** | **4.65%** | **2.2** | **1.70,2.83** | **-12.15%** | **2.01** | **1.64,2.46** | **-12.15%** |
| + Social activity | **2.44** | **2.03,2.94** | **-5.11%** | **2.28** | **1.84,2.82** | **-3.23%** | **1.74** | **1.52,2.00** | **-1.37%** | **1.39** | **1.14,1.68** | **9.30%** | **2.1** | **1.63,2.71** | **-2.80%** | **2** | **1.63,2.45** | **-2.80%** |
| + Physical activity | **2.29** | **1.91,2.75** | **5.84%** | **2.07** | **1.67,2.56** | **13.71%** | **1.71** | **1.49,1.96** | **2.74%** | **1.34** | **1.10,1.62** | **20.93%** | **2.01** | **1.56,2.58** | **5.61%** | **1.96** | **1.60,2.39** | **5.61%** |
| + Personality | **2.21** | **1.84,2.66** | **11.68%** | **2.1** | **1.69,2.59** | **11.29%** | **1.67** | **1.45,1.92** | **8.22%** | **1.39** | **1.15,1.69** | **9.30%** | **2.03** | **1.58,2.62** | **3.74%** | **1.91** | **1.56,2.33** | **3.74%** |
| Fully-adjusted | **2.11** | **1.74,2.56** | **18.98%** | **1.81** | **1.45,2.26** | **34.68%** | **1.62** | **1.40,1.87** | **15.07%** | 1.12 | 0.91,1.38 |  | **2.06** | **1.58,2.70** | **0.93%** | **1.94** | **1.57,2.40** | **0.93%** |
|  |  |  |  |  |  |  |  |  |  |  |  |  |  |  |  |  |  |  |
| ANXIOUS, NOT DEPRESSED | | | | | | | | | | | | | | | | | | |
| Basic model | **1.98** | **1.31,3.00** | **-** | **3.17** | **1.75,5.71** | **-** | **1.73** | **1.26,2.38** | - | **2.31** | **1.35,3.96** | - | **2.15** | **1.15,4.00** | - | **1.77** | **1.12,2.80** | - |
| + Demographics | **1.82** | **1.20,2.76** | **16.33%** | **2.98** | **1.65,5.39** | **8.76%** | **1.69** | **1.23,2.33** | **5.48%** | **1.92** | **1.11,3.31** | **5.48%** | **1.95** | **1.04,3.64** | **5.48%** | **1.7** | **1.07,2.70** | **5.48%** |
| + SES | **1.95** | **1.29,2.95** | **3.06%** | **3.18** | **1.76,5.75** | **-0.46%** | **1.71** | **1.25,2.36** | **2.74%** | **2.15** | **1.24,3.72** | **2.74%** | **2.12** | **1.14,3.97** | **2.74%** | **1.82** | **1.15,2.89** | **2.74%** |
| + Urbanisation | **1.98** | **1.31,3.00** | **0.00%** | **3.16** | **1.75,5.70** | **0.46%** | **1.73** | **1.26,2.38** | **0.00%** | **2.31** | **1.35,3.95** | **0.00%** | **2.14** | **1.15,3.98** | **0.00%** | **1.76** | **1.11,2.78** | **0.00%** |
| + Physical health | **1.97** | **1.30,2.98** | **1.02%** | **3.08** | **1.71,5.57** | **4.15%** | **1.75** | **1.27,2.40** | **-2.74%** | **2.29** | **1.33,3.92** | **-2.74%** | **2.22** | **1.19,4.14** | **-2.74%** | **1.8** | **1.14,2.85** | **-2.74%** |
| + Social activity | **2.03** | **1.34,3.07** | **-5.10%** | **3.22** | **1.78,5.81** | **-2.30%** | **1.73** | **1.26,2.38** | **0.00%** | **2.34** | **1.36,4.01** | **0.00%** | **2.2** | **1.18,4.11** | **0.00%** | **1.79** | **1.13,2.83** | **0.00%** |
| + Physical activity | **1.95** | **1.29,2.95** | **3.06%** | **3.05** | **1.69,5.51** | **5.53%** | **1.72** | **1.25,2.37** | **1.37%** | **2.23** | **1.30,3.83** | **1.37%** | **2.11** | **1.13,3.93** | **1.37%** | **1.77** | **1.12,2.80** | **1.37%** |
| + Personality | **1.71** | **1.13,2.61** | **27.55%** | **2.94** | **1.62,5.33** | **10.60%** | **1.63** | **1.18,2.25** | **13.70%** | **2.24** | **1.30,3.87** | **13.70%** | **2.13** | **1.14,3.99** | **13.70%** | **1.68** | **1.05,2.68** | **13.70%** |
| Fully-adjusted | **1.65** | **1.07,2.52** | **33.67%** | **2.74** | **1.50,5.00** | **19.82%** | **1.59** | **1.15,2.20** | **19.18%** | **1.88** | **1.06,3.32** | **19.18%** | **2.13** | **1.12,4.03** | **19.18%** | **1.74** | **1.09,2.79** | **19.18%** |

*Notes : Boldface=p<.05. PPAE=percentage of protective association explained. Basic model adjusted for frequency of engagement (never, once in last 12 months, twice in last 12 months, 3-4 times in last 12 months). Demographics=age, gender, ethnicity. SES=employment status, educational attainment, household income. Urbanisation=community type. Physical health=chronic illness, chronic pain, problems affecting mobility. Social activity=frequency of meeting up with friends or family. Physical activity=number of days engaged in 30 minutes of moderate or vigorous exercise in the past week. Personality=extraversion, openness, agreeableness, conscientiousness, neuroticism.*

**Supplementary Table 3: Results from logistic regression analyses showing odds of reporting two or more factors that would encourage engagement in artistic hobbies amongst individuals with (i) depression, and (ii) anxiety compared to individuals with neither depression or anxiety**

|  | CAPABILITIES | | | | | | OPPORTUNITIES | | | | | | MOTIVATORS | | | | | |
| --- | --- | --- | --- | --- | --- | --- | --- | --- | --- | --- | --- | --- | --- | --- | --- | --- | --- | --- |
|  | **PSYCHOLOGICAL** | | | **PHYSICAL** | | | **SOCIAL** | | | **PHYSICAL** | | | **AUTOMATIC** | | | **REFLECTIVE** | | |
|  | **OR** | **95% CI** | **PPAE** | **OR** | **95% CI** | **PPAE** | **OR** | **95CI** | **PPAE** | **OR** | **95CI** | **PPAE** | **OR** | **95CI** | **PPAE** | **OR** | **95CI** | **PPAE** |
| DEPRESSED, NOT ANXIOUS | | | | | | | | | | | | | | | | | | |
| Basic model | **1.53** | **1.30,1.80** | **-** | **2.02** | **1.72,2.39** | **-** | **1.42** | **1.21,1.67** | **-** | **1.42** | **1.20,1.68** | **-** | **1.48** | **1.23,1.78** | **-** | **1.36** | **1.15,1.61** | **-** |
| + Demographics | **1.53** | **1.29,1.81** | **0.00%** | **2.03** | **1.72,2.40** | **-0.98%** | **1.43** | **1.21,1.68** | **-2.38%** | **1.39** | **1.17,1.66** | **7.14%** | **1.48** | **1.23,1.78** | **0.00%** | **1.36** | **1.15,1.61** | **0.00%** |
| + SES | **1.44** | **1.22,1.71** | **16.98%** | **1.94** | **1.64,2.30** | **7.84%** | **1.36** | **1.15,1.60** | **14.29%** | **1.27** | **1.07,1.52** | **35.71%** | **1.46** | **1.22,1.76** | **4.17%** | **1.36** | **1.15,1.61** | **0.00%** |
| + Urbanisation | **1.52** | **1.29,1.80** | **1.89%** | **2.02** | **1.71,2.39** | **0.00%** | **1.41** | **1.20,1.66** | **2.38%** | **1.42** | **1.20,1.68** | **0.00%** | **1.48** | **1.23,1.77** | **0.00%** | **1.36** | **1.15,1.61** | **0.00%** |
| + Physical health | **1.52** | **1.29,1.79** | **1.89%** | **1.85** | **1.56,2.19** | **16.67%** | **1.4** | **1.19,1.65** | **4.76%** | **1.37** | **1.16,1.62** | **11.90%** | **1.47** | **1.23,1.77** | **2.08%** | **1.36** | **1.15,1.61** | **0.00%** |
| + Social activity | **1.52** | **1.29,1.80** | **1.89%** | **2** | **1.69,2.36** | **1.96%** | **1.41** | **1.19,1.66** | **2.38%** | **1.38** | **1.17,1.64** | **9.52%** | **1.49** | **1.24,1.79** | **-2.08%** | **1.41** | **1.19,1.67** | **-13.89%** |
| + Physical activity | **1.5** | **1.27,1.77** | **5.66%** | **1.89** | **1.60,2.23** | **12.75%** | **1.41** | **1.19,1.66** | **2.38%** | **1.37** | **1.16,1.63** | **11.90%** | **1.46** | **1.22,1.76** | **4.17%** | **1.37** | **1.16,1.63** | **-2.78%** |
| + Personality | **1.46** | **1.24,1.73** | **13.21%** | **1.93** | **1.63,2.28** | **8.82%** | **1.38** | **1.17,1.63** | **9.52%** | **1.4** | **1.18,1.66** | **4.76%** | **1.47** | **1.22,1.76** | **2.08%** | **1.35** | **1.14,1.60** | **2.78%** |
| Fully-adjusted | **1.37** | **1.15,1.63** | **30.19%** | **1.63** | **1.36,1.95** | **38.24%** | **1.31** | **1.11,1.56** | **26.19%** | 1.19 | 0.99,1.43 |  | **1.45** | **1.20,1.75** | **6.25%** | **1.4** | **1.17,1.67** | **-11.11%** |
|  |  |  |  |  |  |  |  |  |  |  |  |  |  |  |  |  |  |  |
| ANXIOUS, NOT DEPRESSED | | | | | | | | | | | | | | | | | | |
| Basic model | **1.48** | **1.25,1.76** | **-** | **1.96** | **1.66,2.32** | **-** | **1.43** | **1.21,1.69** | **-** | **1.58** | **1.33,1.89** | **-** | **1.46** | **1.21,1.76** | **-** | **1.89** | **1.58,2.27** | **-** |
| + Demographics | **1.41** | **1.18,1.68** | **14.58%** | **1.9** | **1.60,2.26** | **6.25%** | **1.41** | **1.19,1.67** | **4.65%** | **1.42** | **1.19,1.70** | **27.59%** | **1.42** | **1.18,1.72** | **8.70%** | **1.87** | **1.56,2.25** | **2.25%** |
| + SES | **1.44** | **1.21,1.71** | **8.33%** | **1.9** | **1.60,2.26** | **6.25%** | **1.4** | **1.19,1.66** | **6.98%** | **1.46** | **1.22,1.75** | **20.69%** | **1.44** | **1.19,1.74** | **4.35%** | **1.9** | **1.58,2.28** | **-1.12%** |
| + Urbanisation | **1.48** | **1.25,1.76** | **0.00%** | **1.96** | **1.66,2.33** | **0.00%** | **1.43** | **1.21,1.69** | **0.00%** | **1.59** | **1.34,1.90** | **-1.72%** | **1.46** | **1.21,1.76** | **0.00%** | **1.9** | **1.58,2.28** | **-1.12%** |
| + Physical health | **1.47** | **1.24,1.74** | **2.08%** | **1.93** | **1.62,2.29** | **3.13%** | **1.43** | **1.21,1.69** | **0.00%** | **1.55** | **1.30,1.85** | **5.17%** | **1.46** | **1.21,1.76** | **0.00%** | **1.89** | **1.58,2.27** | **0.00%** |
| + Social activity | **1.47** | **1.24,1.75** | **2.08%** | **1.95** | **1.64,2.31** | **1.04%** | **1.43** | **1.21,1.69** | **0.00%** | **1.57** | **1.32,1.87** | **1.72%** | **1.46** | **1.21,1.76** | **0.00%** | **1.9** | **1.58,2.27** | **-1.12%** |
| + Physical activity | **1.48** | **1.24,1.75** | **0.00%** | **1.95** | **1.64,2.31** | **1.04%** | **1.43** | **1.21,1.69** | **0.00%** | **1.57** | **1.32,1.87** | **1.72%** | **1.45** | **1.21,1.75** | **2.17%** | **1.9** | **1.58,2.27** | **-1.12%** |
| + Personality | **1.38** | **1.16,1.65** | **20.83%** | **1.88** | **1.58,2.24** | **8.33%** | **1.41** | **1.19,1.68** | **4.65%** | **1.52** | **1.27,1.82** | **10.34%** | **1.44** | **1.19,1.74** | **4.35%** | **1.82** | **1.51,2.19** | **7.87%** |
| Fully-adjusted | **1.28** | **1.07,1.53** | **41.67%** | **1.69** | **1.40,2.04** | **28.13%** | **1.36** | **1.14,1.62** | **16.28%** | **1.28** | **1.06,1.55** | **51.72%** | **1.4** | **1.15,1.70** | **13.04%** | **1.81** | **1.50,2.19** | **8.99%** |

*Notes : Boldface=p<.05. PPAE=percentage of protective association explained. Basic model adjusted for frequency of engagement (never, once in last 12 months, twice in last 12 months, 3-4 times in last 12 months). Demographics=age, gender, ethnicity. SES=employment status, educational attainment, household income. Urbanisation=community type. Physical health=chronic illness, chronic pain, problems affecting mobility. Social activity=frequency of meeting up with friends or family. Physical activity=number of days engaged in 30 minutes of moderate or vigorous exercise in the past week. Personality=extraversion, openness, agreeableness, conscientiousness, neuroticism.*

**Supplementary Table 4: Results from logistic regression analyses showing odds of reporting three or more factors that would encourage engagement in artistic hobbies amongst individuals with (i) depression, and (ii) anxiety compared to individuals with neither depression or anxiety**

|  | CAPABILITIES | | | | | | OPPORTUNITIES | | | | | | MOTIVATORS | | | | | |
| --- | --- | --- | --- | --- | --- | --- | --- | --- | --- | --- | --- | --- | --- | --- | --- | --- | --- | --- |
|  | **PSYCHOLOGICAL** | | | **PHYSICAL** | | | **SOCIAL** | | | **PHYSICAL** | | | **AUTOMATIC** | | | **REFLECTIVE** | | |
|  | **OR** | **95% CI** | **PPAE** | **OR** | **95% CI** | **PPAE** | **OR** | **95CI** | **PPAE** | **OR** | **95CI** | **PPAE** | **OR** | **95CI** | **PPAE** | **OR** | **95CI** | **PPAE** |
| DEPRESSED, NOT ANXIOUS | | | | | | | | | | | | | | | | | | |
| Basic model | **1.76** | **1.45,2.14** | **-** | **1.53** | **1.20,1.94** | **-** | **1.64** | **1.35,1.99** | **-** | **1.13** | **0.94,1.35** | **-** | **1.51** | **1.29,1.78** | **-** | **1.25** | **1.05,1.48** | **-** |
| + Demographics | **1.77** | **1.46,2.15** | **-1.32%** | **1.58** | **1.24,2.01** | **-9.43%** | **1.68** | **1.38,2.04** | **-6.25%** | **1.1** | **0.92,1.32** | **23.08%** | **1.52** | **1.29,1.79** | **-1.96%** | **1.25** | **1.05,1.48** | **0.00%** |
| + SES | **1.62** | **1.33,1.97** | **18.42%** | **1.44** | **1.13,1.84** | **16.98%** | **1.59** | **1.31,1.94** | **7.81%** | **0.99** | **0.82,1.19** | **107.69%** | **1.49** | **1.26,1.76** | **3.92%** | **1.23** | **1.04,1.47** | **8.00%** |
| + Urbanisation | **1.75** | **1.45,2.13** | **1.32%** | **1.54** | **1.21,1.96** | **-1.89%** | **1.63** | **1.34,1.98** | **1.56%** | **1.13** | **0.94,1.35** | **0.00%** | **1.51** | **1.28,1.78** | **0.00%** | **1.25** | **1.05,1.48** | **0.00%** |
| + Physical health | **1.7** | **1.40,2.07** | **7.89%** | **1.23** | **0.95,1.59** | **56.60%** | **1.6** | **1.31,1.94** | **6.25%** | **1.1** | **0.92,1.32** | **23.08%** | **1.5** | **1.27,1.77** | **1.96%** | **1.25** | **1.05,1.48** | **0.00%** |
| + Social activity | **1.75** | **1.44,2.13** | **1.32%** | **1.49** | **1.17,1.89** | **7.55%** | **1.6** | **1.31,1.94** | **6.25%** | **1.1** | **0.92,1.31** | **23.08%** | **1.53** | **1.30,1.80** | **-3.92%** | **1.25** | **1.05,1.48** | **0.00%** |
| + Physical activity | **1.71** | **1.41,2.08** | **6.58%** | **1.45** | **1.14,1.85** | **15.09%** | **1.62** | **1.34,1.97** | **3.12%** | **1.11** | **0.92,1.32** | **15.38%** | **1.48** | **1.26,1.75** | **5.88%** | **1.25** | **1.06,1.49** | **0.00%** |
| + Personality | **1.66** | **1.36,2.02** | **13.16%** | **1.5** | **1.18,1.92** | **5.66%** | **1.59** | **1.31,1.93** | **7.81%** | **1.1** | **0.92,1.32** | **23.08%** | **1.51** | **1.28,1.78** | **0.00%** | **1.27** | **1.07,1.51** | **-8.00%** |
| Fully-adjusted | **1.5** | **1.22,1.84** | **34.21%** | **1.14** | **0.87,1.49** | **73.58%** | **1.52** | **1.24,1.87** | **18.75%** | 0.93 | 0.77,1.13 |  | **1.48** | **1.25,1.76** | **5.88%** | **1.26** | **1.05,1.50** | **-4.00%** |
|  |  |  |  |  |  |  |  |  |  |  |  |  |  |  |  |  |  |  |
| ANXIOUS, NOT DEPRESSED | | | | | | | | | | | | | | | | | | |
| Basic model | **1.94** | **1.60,2.36** | **-** | **1.45** | **1.13,1.86** | **-** | **1.73** | **1.42,2.11** | **-** | **1.43** | **1.20,1.71** | **-** | **1.66** | **1.40,1.96** | **-** | **1.55** | **1.31,1.85** | **-** |
| + Demographics | **1.91** | **1.57,2.33** | **3.19%** | **1.56** | **1.21,2.02** | **-24.44%** | **1.79** | **1.46,2.19** | **-8.22%** | **1.3** | **1.08,1.56** | **30.23%** | **1.65** | **1.39,1.95** | **1.52%** | **1.54** | **1.29,1.83** | **1.82%** |
| + SES | **1.88** | **1.54,2.29** | **6.38%** | **1.44** | **1.12,1.85** | **2.22%** | **1.71** | **1.40,2.09** | **2.74%** | **1.31** | **1.09,1.57** | **27.91%** | **1.63** | **1.38,1.93** | **4.55%** | **1.53** | **1.29,1.82** | **3.64%** |
| + Urbanisation | **1.94** | **1.60,2.36** | **0.00%** | **1.45** | **1.13,1.86** | **0.00%** | **1.73** | **1.42,2.10** | **0.00%** | **1.43** | **1.20,1.71** | **0.00%** | **1.66** | **1.40,1.97** | **0.00%** | **1.55** | **1.31,1.85** | **0.00%** |
| + Physical health | **1.91** | **1.57,2.32** | **3.19%** | **1.37** | **1.05,1.79** | **17.78%** | **1.71** | **1.40,2.09** | **2.74%** | **1.41** | **1.18,1.69** | **4.65%** | **1.64** | **1.39,1.94** | **3.03%** | **1.55** | **1.31,1.85** | **0.00%** |
| + Social activity | **1.94** | **1.59,2.36** | **0.00%** | **1.46** | **1.14,1.87** | **-2.22%** | **1.73** | **1.42,2.10** | **0.00%** | **1.42** | **1.19,1.70** | **2.33%** | **1.66** | **1.40,1.97** | **0.00%** | **1.55** | **1.30,1.84** | **0.00%** |
| + Physical activity | **1.93** | **1.59,2.34** | **1.06%** | **1.43** | **1.12,1.84** | **4.44%** | **1.73** | **1.42,2.10** | **0.00%** | **1.42** | **1.19,1.70** | **2.33%** | **1.65** | **1.39,1.95** | **1.52%** | **1.55** | **1.31,1.85** | **0.00%** |
| + Personality | **1.79** | **1.46,2.19** | **15.96%** | **1.49** | **1.15,1.93** | **-8.89%** | **1.69** | **1.38,2.07** | **5.48%** | **1.38** | **1.15,1.65** | **11.63%** | **1.64** | **1.38,1.95** | **3.03%** | **1.59** | **1.33,1.90** | **-7.27%** |
| Fully-adjusted | **1.7** | **1.38,2.10** | **25.53%** | **1.36** | **1.03,1.81** | **20.00%** | **1.66** | **1.35,2.05** | **9.59%** | 1.18 | 0.98,1.44 |  | **1.59** | **1.33,1.90** | **10.61%** | **1.55** | **1.29,1.87** | **0.00%** |

*Notes : Boldface=p<.05. PPAE=percentage of protective association explained. Basic model adjusted for frequency of engagement (never, once in last 12 months, twice in last 12 months, 3-4 times in last 12 months). Demographics=age, gender, ethnicity. SES=employment status, educational attainment, household income. Urbanisation=community type. Physical health=chronic illness, chronic pain, problems affecting mobility. Social activity=frequency of meeting up with friends or family. Physical activity=number of days engaged in 30 minutes of moderate or vigorous exercise in the past week. Personality=extraversion, openness, agreeableness, conscientiousness, neuroticism*
